# Continuous Magnesium Sulfate Infusions for Status Asthmaticus in Children: A Systematic Review

**DOI:** 10.3389/fped.2022.853574

**Published:** 2022-03-22

**Authors:** Peter N. Johnson, Anna Sahlstrom Drury, Neha Gupta

**Affiliations:** ^1^Department of Pharmacy, Clinical and Administrative Sciences, University of Oklahoma College of Pharmacy, Oklahoma City, OK, United States; ^2^Department of Pharmacy, University of Kentucky Chandler Medical Center, Lexington, KY, United States; ^3^Division of Critical Care Medicine, Department of Pediatrics, University of Oklahoma College of Medicine, Oklahoma City, OK, United States

**Keywords:** magnesium, infusion, status asthmaticus, children, pediatric intensive care unit

## Abstract

**Objectives:**

Magnesium sulfate is a second-tier therapy for asthma exacerbations in children; guidelines recommend a single-dose to improve pulmonary function and decrease the odds of admission to the in-patient setting. However, many clinicians utilize prolonged magnesium sulfate infusions for children with refractory asthma. The purpose of this review is to describe the efficacy and safety of magnesium sulfate infusions administered over ≥ 1 h in children with status asthmaticus.

**Methods:**

Medline was searched using the keywords “magnesium sulfate” and “children.” Articles evaluating the use of magnesium sulfate infusions for ≥1 h published between 1946 and August 2021 were included. Published abstracts were not included because of lack of essential details. All articles were screened by two reviewers.

**Results:**

Eight reports including 447 children were included. The magnesium regimens evaluated included magnesium delivered over 1 h (*n* = 148; 33.1%), over 4–5 h (*n* = 105; 23.5%), and over >24 h (*n* = 194; 43.4%). Majority of patients received a bolus dose of 25–75 mg/kg/dose prior to initiation of a prolonged infusion (*n* = 299; 66.9%). For the patients receiving magnesium infusions over 4–5 h, the dosing regimen varied between 40 and 50 mg/kg/h. For those receiving magnesium infusions >24 h, the dosing varied between 18.4 and 25 mg/kg/h for a duration between 53.4 and 177.5 h. Only three reports including 186 patients (41.6%) included an evaluation of clinical outcomes including evaluation of lung function parameters, reduction in PICU transfers, and/or decrease in emergency department length of stay. Five reports including 261 patients (58.4%) evaluated magnesium serum concentrations. In most reports, the goal concentrations were between 4 and 6 mg/dL. Only 3 (1.1%) out of the 261 patients had supratherapeutic magnesium concentrations. The only reports finding adverse events attributed to magnesium were noted in those receiving infusions for >24 h. Clinically significant adverse events included hypotension (*n* = 74; 16.6%), nausea/vomiting (*n* = 35; 7.8%), mild muscle weakness (*n* = 22; 4.9%), flushing (*n* = 10; 2.2%), and sedation (*n* = 2; 0.4%).

**Conclusion:**

Significant variability was noted in magnesium dosing regimens, with most children receiving magnesium infusions over >4 h. Most reports did not assess clinical outcomes. Until future research is conducted, the use of prolonged magnesium sulfate infusions should be reserved for refractory asthma therapy.

## Introduction

In the United States of America, approximately 7.1 million children have asthma, and these children experience approximately 680,000 emergency department (ED) visits and >70,000 hospitalizations annually (1). Among children admitted to the pediatric intensive care unit (PICU), those with severe asthma exacerbations, also referred to as status asthmaticus or critical asthma, have increased morbidity and associated health-care costs compared to those with mild to moderate asthma exacerbations (2). Several organizations like the National Asthma Education and Prevention Program Coordinating Committee (NAEPPCC) and Global Initiative for Asthma (GINA) have published guidelines on the management of children with asthma exacerbations (3–5). However, these guidelines provide limited recommendations for children with status asthmaticus or critical asthma. In these patients, the standard of care includes intravenous (IV) corticosteroids and nebulized continuous short-acting beta-2-agonists (SABA). However, providers have utilized several second-tier pharmacologic therapies including heliox, IV aminophylline, IV ketamine, and IV terbutaline (6).

Another therapy that has been proposed as an option for a second tier is the use of IV magnesium sulfate infusions. The NAEPPCC and GINA guidelines provide recommendations for a single dose of IV magnesium sulfate in children with asthma exacerbation in the ED with refractory clinical manifestations 1 h after receipt of oral/IV corticosteroids and repeated doses of SABAs (4, 5). The dosing regimen recommended is between 25 and 75 mg/kg with a maximum of 2 g/dose over 20 min (4, 7). This regimen is associated with an improvement in pulmonary function and 68% decreased odds of admission to the hospital when administered in the ED setting (8, 9). However, some sources have provided recommendations on the use of continuous magnesium sulfate infusions in the ED or PICU setting for children with refractory status asthmaticus (6). The purpose of this review is to describe the efficacy and safety of magnesium sulfate infusions in children with asthma exacerbations or status asthmaticus who received magnesium sulfate infusions administered over ≥1 h.

## Materials and Methods

Relevant articles were identified from Medline (1946–August 2021) using the terms “magnesium sulfate” and “children.” Results were limited to studies in humans. Published abstracts were not included because of lack of essential details. Thus, the search was limited to published studies. To be included, the reports had to include children receiving a magnesium IV infusion administered over ≥1 h for asthma exacerbations refractory to common treatments and had to be published in the English language.

## Results

**FIGURE 1:**
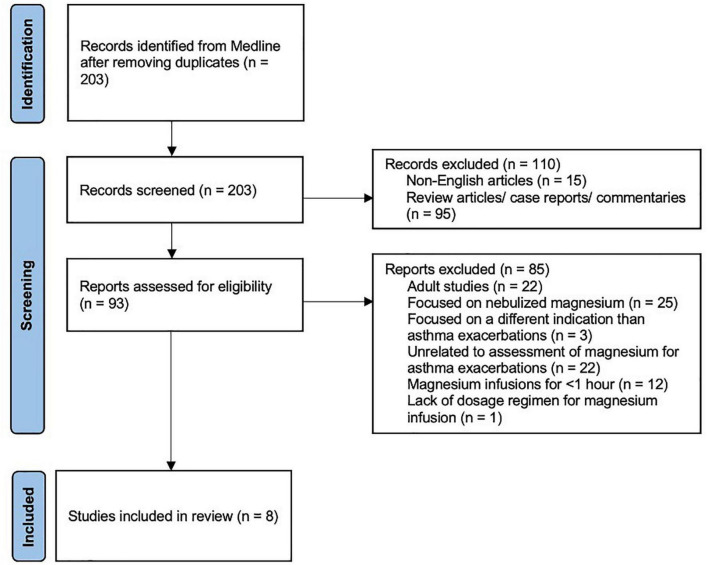
PRISMA flow diagram of included studies.

The PRISMA diagram of the included studies is shown in Figure 1. The electronic search identified 203 reports for title and abstract review that were imported into Convidence^®^. All articles were screened by two reviewers, and all authors were involved in the final selection process. One hundred ten records were excluded as they were either non-English articles or were review articles or case reports or commentaries. Ninety-three full-text studies were assessed for eligibility. Eighty-five studies were excluded for several reasons including studies done in the adult population, focused on nebulized magnesium, or involved administration of magnesium sulfate over <1 h. One report evaluated the use of a prolonged magnesium sulfate infusion for refractory asthma exacerbation, but they did not provide clear details on their dosing regimen; therefore, it was excluded from further review (10) (Figure 1).

A total of eight reports including 447 children were included (11–18). These eight reports included evaluation of different approaches to magnesium infusions. Two of the reports discussed a magnesium infusion administered over a 1-h period (11, 12). Four reports evaluated the efficacy and safety of magnesium infusions administered over 4–5 h (13–16). The remaining two reports described the use of magnesium infusions administered over >24 h (17, 18). Table 1 provides an overview of these reports including the type of report, demographics, place in therapy, dosage regimen, and main outcomes.

**TABLE 1:** Overview of reports evaluating the use of magnesium infusions.

Reference (study type)	Sample size	Age (years)	Place in therapy for magnesium infusions	Magnesium dosing regimen (bolus/infusion and dosing)	Magnesium infusion duration	Results
**Magnesium infusion administered over 1 h**						
DeSanti et al. (11) (Retrospective matched cohort analysis)	Magnesium group (*n* = 33); control group (*n* = 33)	2–18 years (specific age not reported); 27 (81.8%) < 10 years	Added after patients received three doses of albuterol 2.5 mg with ipratropium 0.5 mg and systemic corticosteroids and at least 6 h of continuous albuterol 0.5 mg/kg/h	*Bolus*: None *Infusion*: >1 dose of 25–75 mg/kg/dose (standard 50 mg/kg/dose); max 2,000 mg/dose	1 h	Patients receiving magnesium had longer median duration of continuous albuterol than controls (34 versus 18 h; *p* = 0.001) and longer length of stay (72 versus 49 h; *p* = 0.037). More patients in magnesium group transferred to PICU compared to controls (9 versus 2 patients, *p* = 0.065). No significant difference in ADEs in each group; both had patients with hypotension (*n* = 3 magnesium; *n* = 3 controls) and respiratory depression (*n* = 8 magnesium; *n* = 3 controls)
Özdemir and Doðruel (12) (Open intervention study)	115	6–17 years (specific age not reported)	Included patients with no SABA use in past 3 h, no oral/IV steroids in last 12 h who were on room air. FEV1 was between 40 and 75% of predicted FEV1	*Bolus*: None *Infusion*: 40–50 mg/kg × 1 dose (max 1,500 mg for patients >30 kg)	1 h	Lung function parameters (FEV1/FVC ratio, FEV1, PEF, FEF_25–75_) pre and post treatment showed statistically significant improvement with the magnesium infusion in children with mild and moderate asthma exacerbations. Mean change in FEV1 with magnesium infusion was 7.7% in the mild group and 10.9% in the moderate group
**Magnesium infusions administered over 4–5 h**						
Irazuzta et al. (13) (Retrospective chart review)	19	Mean 9.3 ± 4.6 years	Administered after nebulized albuterol, IV corticosteroids, two doses of nebulized ipratropium, and one dose of IV magnesium sulfate in the ED	*Bolus*: 50 mg/kg in ED; followed by 75 mg/kg (≤30kg) or 50 mg/kg (> 30kg) over 30–45 min *Infusion*: 40 mg/kg/h for 4 h	4 h	Serum magnesium concentrations at end of infusion were 4.4 ± 0.8 mg/dL Three patients noted discomfort with the infusions but received them as ordered. No patients needed to discontinue the infusion due to ADEs. No episodes of hypotension, respiratory failure, neurologic problems, or nausea
Egelund et al. (14) (Prospective cohort study)	Magnesium group (*n* = 19); control group (*n* = 38)	Mean 8.9 ± 4.2	Included patients admitted to the PICU with status asthmaticus	*Bolus*: one dose of 75 mg/kg (≤30 kg) or 50 mg/kg (> 30kg) over 30–45 min *Infusion*: 40 mg/kg/h infusion (utilized ideal body weight if body mass index >30 kg/m^2^)	4 h	Three patients had mild infusion-related reactions with magnesium, but no significant ADEs were noted. No difference between groups in systolic and diastolic blood pressure or oxygen saturation. Heart rate and respiratory rate were lower in the magnesium group (heart rate *p* = 0.03, respiratory rate *p* = 0.01) but not clinically significant. Mean serum magnesium concentration post infusion was 4.4 ± 0.98 mg/dL
Vaiyani and Irazuzta (15) (Retrospective study comparing two magnesium infusion regimens)	Standard high-dose infusion group (*n* = 19); simplified infusion group (*n* = 10)	1–17 years (specific age not reported)	Administered after IV corticosteroids, two doses of nebulized ipratropium, and 5 mg of nebulized salbutamol every 20 min after 2 h of treatment	Standard high-dose infusion: *Bolus*: ≤30 kg—75 mg/kg or >30kg—50 mg/kg over 30–45 min *Infusion*: 40 mg/kg/h for 4 h (utilized ideal body weight if body mass index > 30 kg/m^2^) Simplified infusion group: *Bolus*: None *Infusion*: 50 mg/kg/h for 5 h	4–5 h (depending on group)	No significant difference in magnesium concentration between groups. No significant difference in hemodynamic parameters, oxygen saturation, or respiratory rate between groups. No significant difference in ADEs between groups
Irazuzta et al. (16) (Prospective, randomized, open-label study)	Magnesium prolonged bolus group (*n* = 19); High-dose magnesium infusion group (n = 19)	Magnesium prolonged bolus: 9.0 ± 2.9 years; High-dose magnesium infusion: 11.1 ± 3.8 years	Administered after IV corticosteroids and 5 mg of nebulized salbutamol every 20 min for 2 h	Prolonged magnesium bolus: *Bolus*:50 mg/kg bolus over 1 h High-dose magnesium infusion: *Bolus*: None *Infusion*: 50 mg/kg/h (max 8,000 mg) for 4 h	1 h (prolonged bolus group) 4 h (high-dose infusion group)	More patients discharged from ED within 24 h in the infusion versus bolus group, 47 versus 10%, *p* = 0.032; with absolute risk reduction of 37% (95% CI: 10–63%). Total length of stay was lower in the infusion versus bolus group, 34.13 ± 19.54 h versus 48.05 ± 18.72 h, *p* = 0.013. No ADEs noted in either group
**Magnesium infusion administered over** > **24 h**						
Glover et al. (17) (Retrospective chart review)	40	Mean 6.8 ± 5.4 years	Administered after nebulized albuterol, ipratropium bromide, and IV methylprednisolone; 28 patients received aminophylline and four patients received ketamine	*Bolus*: Administered in 21 patients; overall mean 29.6 ± 13.2 mg/kg; administration time not defined *Infusion*: Overall mean 18.4 ± 6.5 mg/kg/h	Overall mean: 75.2 ± 74.9 h ≤30 kg: 93.8 ± 89.2 h >30 kg: 49.9 ± 39.3 h	Significant difference between those ≤30 kg versus >30 kg for initial bolus dose, 35.3 ± 12.7 versus 21.9 ± 12.7 mg/kg (*p* < 0.05) and maintenance infusion, 21.6 ± 6.0 versus 14.6 ± 4.2 mg/kg/h (*p* < 0.05). No difference in serum magnesium concentrations between those ≤30 kg versus >30 kg, 3.9 ± 0.6 versus 3.6 ± 0.5 mg/dL (*p* > 0.05). No cardiovascular ADEs noted with magnesium; one magnesium infusion was stopped in a patient who was experiencing over-sedation
Graff et al. (18) (Retrospective chart review)	154	Median 8 years (IQR 5–11.8 years)	Nebulized albuterol continuously or every 2 h, ipratropium bromide, and systemic corticosteroids; 40 patients received adjunctive therapies (aminophylline, terbutaline, and/or theophylline) while on the magnesium infusion	*Bolus*: 50–70 mg/kg (max 2,000 mg/dose) over 20 min *Infusion*: 25 mg/kg/h (max 2,000 mg/h), titrated in 5 mg/kg/h increments to maintain serum magnesium concentration between 4 and 6 mg/dL (i.e., drawn every 6 h during infusion)	Median 53.4 h (range 24–177.5 h)	82.5% of patients reached the therapeutic range by the 2nd concentration and 95% by the 3rd concentration. 48.1% of patients experienced hypotension that was primarily diastolic hypotension (94%). Five patients with hypotension events required interventions: 0.9% saline bolus (*n* = 2), maintenance IV fluids (*n* = 1), and reduction in magnesium infusion rate (*n* = 2). Nine of those without hypotension also received fluid boluses. Non-cardiovascular non-severe ADEs included nausea/emesis (22.7%), transient weakness (14.9%), flushing (6.5%); severe ADEs included hypotonia (0.65%), escalation of respiratory therapy (1.9%), and sedation (0.65%). Supratherapeutic concentrations >6 mg/dL occurred in 2% of patients and not associated with ADEs

*ADEs, adverse drug events; SABA, short acting beta agonist; IV, intravenous; FEV_1_, forced expiratory volume in 1 s; FVC, forced vital capacity; PEF, peak expiratory flow; FEF_25–75_, forced expiratory flow at 25–75% of forced vital capacity; ED, emergency department; PICU, pediatric intensive care unit; IQR, interquartile range.*

### Magnesium Infusions Administered Over 1 h

DeSanti et al. conducted a retrospective matched cohort analysis in children admitted to a non-intensive care setting on continuous albuterol therapy (11). The goal of this study was to determine the effect of magnesium sulfate on the duration of continuous albuterol and hospital length of stay (LOS). They compared 33 patients receiving intravenous (IV) magnesium sulfate infusion versus a control group (*n* = 33) with similar respiratory assessment scores. The IV magnesium sulfate doses ranged from 25 to 75 mg/kg and were administered over 60 min. Four patients in each group received magnesium sulfate in the ED prior to being admitted to the hospital. The authors noted that patients who received IV magnesium sulfate once admitted, had longer durations of continuous albuterol therapy (*p* = 0.001) and a longer hospital LOS (*p* = 0.037). In the magnesium sulfate group, the authors found no significant difference in adverse reactions including hypotension and respiratory depression between groups; the authors noted that neither of these adverse events were directly attributed to magnesium. They concluded that those who received magnesium sulfate did not have reduced continuous albuterol therapy duration or LOS. It is important to note that the patients’ respiratory status was determined using a non-validated tool, limiting the external validity of their findings. Compared with other studies, they initiated magnesium sulfate later in the course of therapy, and they did not monitor magnesium sulfate concentrations, which may have been subtherapeutic.

Özdemir and colleagues conducted an open intervention study in children presenting to a pediatric pulmonary clinic with mild (Group 1) (*n* = 50) to moderate (Group 2) (*n* = 65) asthma to determine the effects of IV magnesium sulfate on patients’ spirometry values (12). All patients received a 40–50 mg/kg IV infusion (maximum 1,500 mg/dose for patients >30 kg) of magnesium sulfate over 60 min. Fifteen minutes after the magnesium sulfate infusion, both mild and moderate asthma groups showed statistically significant increases in forced expiratory volume in 1 s over forced vital capacity (FEV_1_/FVC), FEV_1_, peak expiratory flow (PEF), and forced expiratory flow at 25–75% of forced vital capacity (FEF_25–75_) (*p* < 0.01). There were no statistically significant differences in oxygen saturation, heart rate, and blood pressure before and after treatment with magnesium sulfate. The authors concluded that IV magnesium sulfate could aid spirometric parameters in children presenting with acute asthma without many adverse events. The authors did not report the number of patients that needed additional asthma therapy after IV magnesium sulfate administration, and they did not include a control group in their study. Additionally, patients were only included in the study if they had oxygen saturations >92% on room air.

### Magnesium Infusions Administered Over 4–5 h

Irazuzta et al. conducted a retrospective study over a 3-year period evaluating the feasibility of a 4-h magnesium infusion protocol in 19 children with status asthmaticus (13). These patients had failed to improve with conventional therapy including at least one dose of magnesium sulfate 50 mg/kg IV in the ED. Their magnesium infusion protocol in the PICU consisted of an IV magnesium sulfate loading dose of 75 mg/kg if ≤30 kg or 50 mg/kg if >30 kg over 30–40 min followed by a 40 mg/kg/h infusion for 4 h. Patients that were mechanically ventilated or received non-invasive ventilation were not eligible to receive magnesium infusions. During the infusion, none of the patients discontinued magnesium sulfate infusion due to adverse events, and there were no reported symptoms of hypotension, flushing, or nausea and vomiting. Twelve (63.2%) patients had serum concentrations of magnesium and electrocardiograms performed; both of which were within normal limits. By the end of the infusion, serum magnesium concentrations were 4.4 ± 0.8 mg/dL and ionized magnesium concentrations were 0.95 ± 0.2 mmol/L. The predictive value of serum magnesium and ionized magnesium concentrations was only moderate, with r^2^ = 0.541. The authors concluded that magnesium infusions were feasible in the PICU, and serum magnesium concentrations did not predict ionized magnesium concentrations. This study was limited in that it was a retrospective study, so it is difficult to determine the timing of the magnesium sulfate infusion and adverse events. In addition, these findings lacked an assessment of clinical outcomes.

Egelund et al. performed a follow-up prospective cohort study of the report by Irazuzta et al. in 57 patients admitted to the PICU with status asthmaticus (13, 14). They compared the safety of magnesium sulfate infusion in 19 children receiving a magnesium infusion versus 38 children in the control group. In addition, they utilized magnesium concentrations to determine the pharmacokinetic parameters of patients receiving magnesium sulfate. Patients that had an instrumented airway or tracheotomy or history of renal dysfunction were not eligible for inclusion in the study. The patients in the treatment group received the same magnesium infusion regimen as in the initial study by Irazuzta et al. (13). However, in this study, the magnesium doses for patients with a body mass index (BMI) >30 kg/m^2^ was based on their ideal body weight. They compared vital signs and magnesium concentrations before bolus, after bolus, mid-infusion, and at the end of the infusion. There was a significant difference in the age between the magnesium infusion and control groups (*p* = 0.0038); no other differences in demographics were noted. There were statistically significant differences between treatment and control groups in heart rate (-5.95 beats per min, 95% CI: -11 to -0.73; *p* = 0.03) and respiratory rate (-4.69 breaths per min, 95% CI: -7.79 to -1.60; *p* = 0.01), but these results were not clinically significant. In addition, there were no significant differences in systolic and diastolic blood pressures and oxygen saturations between groups. Three patients in the treatment group (15.8%) experienced nausea, vomiting, flushing, and injection site pain that the authors attributed to magnesium sulfate therapy. The mean serum magnesium concentration at the end of infusion was 4.4 ± 0.98 mg/dL, and the estimated volume of distribution was 0.4 ± 0.13 L/kg with a clearance of 1.58 ± 0.24 mL/kg/min. The authors noted that magnesium was associated with a lower heart rate and respiratory rate than controls, but no significant adverse events were noted. They also commented that the serum concentrations at the end of infusion were within the range to achieve smooth muscle relaxation based on data from previous studies. This study was limited in that there was no description of matching performed for the control and treatment groups, and no comparison of clinical outcomes was performed between groups.

As a follow up to the initial feasibility study, Vaiyani et al. conducted a retrospective study in children comparing vital signs and magnesium concentrations between patients receiving two different magnesium infusion regimens in the ED setting (15). Patients with a history of renal dysfunction or chronic respiratory compromise or instrumented airway were excluded from the magnesium infusion protocols. They compared patients receiving the magnesium sulfate regimen from their standard high-dose infusion utilized in previous studies, which consisted of magnesium sulfate IV bolus of 75 mg/kg if ≤30 kg or 50 mg/kg if >30 kg over 30–40 min followed by a 40 mg/kg/h infusion for 4 h (*n* = 19) versus a simplified infusion of 50 mg/kg/h for 5 h with no initial bolus (*n* = 10) (13, 14); for obese patients, magnesium sulfate dosing was determined using ideal body weight. The authors found no significant difference in vital signs or serum magnesium concentrations between groups. No adverse events were noted, and no patients had their magnesium infusion discontinued. The authors determined that patients receiving the simplified magnesium infusion without a loading dose produced similar magnesium concentrations compared to their standard high-dose magnesium infusions with loading doses. They postulated that a simplified dosing regimen could reduce potential for medication errors without changing serum magnesium concentrations. However, it should be noted that this study’s findings are limited by its retrospective design and lack of comparison of clinical outcomes between groups.

Irazutzta et al. conducted a prospective study comparing a prolonged magnesium sulfate bolus versus a high dose simplified magnesium infusion in children 6–16 years of age with status asthmaticus on the rate of patient discharges from the ED at 24 h, LOS, and healthcare costs (16). Patients in this study were randomized to receive either a prolonged IV magnesium sulfate bolus of 50 mg/kg over 1 h (*n* = 19) or a high-dose IV magnesium infusion of 50 mg/kg/h for 4 h (maximum 8,000 mg/4 h) (*n* = 19). Along with the magnesium therapy, patients received standardized additional management strategies including supplemental oxygen via Venturi or rebreathing masks for a goal oxygen saturation of >90%, nebulized albuterol every 2 h, and IV dexamethasone 0.2 mg/kg every 6 h. As patients clinically improved, they were transitioned to oral prednisone 2 mg/kg/dose (maximum 60 mg/dose) every 8 h, nebulized albuterol every 4 h, and switched to nasal canula as their oxygen requirements decreased. Compared to the prolonged bolus group, the high-dose magnesium infusion group had a statistically significant greater chance of being discharged from the ED within 24 h (*p* = 0.032) and a shorter hospital LOS (*p* = 0.013). Additionally, the hospital cost per patient was significantly different between the high-dose infusion and prolonged bolus groups ($603.16 ± 338.47 versus $834.37 ± 306.73 respectively; *p* < 0.016). Similar to their previous studies, there were no clinical hypotension events, and no patient in the study needed to discontinue magnesium sulfate due to adverse events. No patients in the study needed PICU admission or mechanical ventilation. The authors concluded that, for patients with asthma exacerbations unresponsive to conventional therapies alone, high-dose magnesium infusions are superior as adjunctive therapy compared to a prolonged IV bolus of magnesium. Compared to the previous studies, this study did not include an assessment of magnesium concentrations, and there was no description if the magnesium dosing was adjusted for patients who were obese.

### Magnesium Infusions Administered Over >24 h

Glover et al. conducted a retrospective chart review of 40 children who presented with refractory wheezing that received a magnesium sulfate infusion in the PICU (17). The goal of the study was to identify dosing strategies and the safety profile of magnesium. Fifteen patients (36.6%) were mechanically ventilated before receiving magnesium infusions. Twenty-one patients (52.5%) received a mean IV magnesium sulfate dose of 29.6 + 13.2 mg/kg (time of administration not provided), and there was a significant difference in the bolus dose between children ≤30 kg versus >30 kg (*p* < 0.05) (Table 1). The overall mean infusion dose was 18.4 ± 6.5 mg/kg/h, and there was a significant difference in the infusion dose between groups, *p* < 0.05 (Table 1). There was also a significant difference between the duration of magnesium infusions between those ≤30 versus >30 kg (*p* < 0.05) (Table 1). Despite the differences in the dosing between groups, there was no significant differences between magnesium serum concentrations between those ≤30 versus >30 kg (3.9 ± 0.6 versus 3.6 ± 0.5 mg/dL, *p* > 0.05) (Table 1). There were no adverse cardiovascular events during magnesium therapy; one patient had their magnesium infusion discontinued secondary to over-sedation which they attributed to magnesium. The authors concluded that magnesium sulfate infusions are safe in pediatric patients and could be an option for refractory asthma treatment in the PICU. It is important to note that the authors did not assess the impact on clinical outcomes. In addition, the authors did not report when magnesium concentrations were obtained during therapy, so it may be difficult to compare these findings to other studies assessing magnesium concentrations.

Graff et al. performed a retrospective study of 154 children who received magnesium sulfate infusions for >24 h for the treatment of refractory status asthmaticus in the PICU; their primary focus was evaluation of non-cardiac and cardiac adverse events and supratherapeutic magnesium concentrations (18). Their magnesium sulfate infusion regimen included an IV bolus of 50–70 mg/kg (maximum 2,000 mg/dose) over 20 min followed by an infusion at 25 mg/kg/h (maximum 2,000 mg/h); their infusion was titrated by 5 mg/kg/h to obtain magnesium concentrations between 4 and 6 mg/dL. Forty patients (26.0%) received additional adjunctive agents with their magnesium infusion including terbutaline, aminophylline, and/or theophylline. The mean duration of therapy was 53.4 h, and a mean of 7 (range 4–10) magnesium concentrations per patient were obtained during their magnesium infusion. Supratherapeutic concentrations (>6 mg/dL) occurred in 2% of patients and were not associated with adverse events. In terms of safety, there were 170 hypotensive events in 74 patients (48.1%), of which the majority (94%) had primarily diastolic hypotension on one blood pressure reading. Only five hypotensive events required interventions. The authors did not find a significant difference in development of hypotension between patients who received magnesium infusions alone and those who received magnesium infusions and other adjunctive agents (*p* = 0.08). In addition, they did not find a difference in hypotensive events among those who had supratherapeutic concentrations, therapeutic concentrations, or around times of infusion initiation/changes (*p* = 0.57). They noted other non-cardiac adverse events including nausea/emesis (22.7%), transient weakness (14.9%), and flushing (6.5%). Five patients (3.2%) experienced severe adverse events such as hypotonia (0.65%), escalation to continuous or bilevel positive pressure (1.9%), and sedation (0.65%); all of these were attributed to the patient’s underlying condition and not their magnesium infusion. No patient required endotracheal intubation. The authors concluded that magnesium infusions were well tolerated; they did note that diastolic hypotension was common but only a few patients required interventions. While the authors concluded that magnesium is safe, they did not study its efficacy or place in acute asthma therapy.

## Discussion

The use of a short magnesium sulfate infusion administered over 20 min is a common option for children with asthma exacerbations to improve lung function and decrease the odds of hospital admission (4, 7–9). However, many clinicians have opted to utilize longer infusions of magnesium sulfate over ≥1 h for children with refractory asthma exacerbations or status asthmaticus who fail conventional treatments. As noted in our systematic review, there was wide variability in the dosage regimens utilized. The majority (*n* = 299; 66.9%) received magnesium sulfate infusions ≥4 h, with 43.4% of them receiving them >24 h (13–18). Most of these patients (*n* = 261; 58.4%) received these infusions in the PICU, with the remaining patients receiving these agents in the pulmonary clinic, ED, or the in-patient wards (13, 14, 17, 18). Only three reports including 186 patients (41.6%) documented the impact of magnesium infusions on clinical outcomes including lung function parameters or PICU transfers (11, 12, 16).

There was significant variability in the dosing regimens utilized in these reports. Approximately 33.1% of patients received a magnesium sulfate infusion over 1 h with the majority receiving 40–50 mg/kg/dose (11, 12). This dosage regimen is consistent with previous studies that have evaluated the simulated pharmacokinetics of different magnesium sulfate bolus doses. Rower et al. simulated the pharmacokinetics of 54 children receiving magnesium sulfate 50 mg/kg (maximum 2,000 mg/dose) over 20 min to determine the dosage regimen to achieve target magnesium concentrations between 2.5 and 4.0 mg/dL (7). They found that doses between 50 and 75 mg/kg were necessary to achieve serum magnesium concentrations within their targeted range. However, given this study assessed the pharmacokinetics of magnesium sulfate administered over 20 min, it is difficult to elucidate the impact of magnesium sulfate regimens administered ≥1 h like the studies by DeSanti and Özdemir et al. (11, 12).

For the remaining 66.9% (*n* = 299) of patients who received a magnesium infusion over ≥4 h, the majority of these patients (*n* = 251; 56.2%) received a bolus dose of 50–75 mg/kg/dose prior to receiving their subsequent magnesium infusion (13–18). In four reports, patients received 40–50 mg/kg/h of magnesium sulfate over 4–5 h infusion (13–16). Whereas, in two reports assessing magnesium infusions >24 h, patients received 18.4–25 mg/kg/h for a duration of 53.4–177.5 h (17, 18). Only two of these studies compared clinical outcomes and adverse events among patients who received different dosage regimens (15, 16). Given the different dosing regimens and study designs of these reports, it is difficult to compare these studies.

Two studies reported that patients’ doses were determined by ideal body weight rather than actual body weight, but they did not articulate any clinical differences in non-obese versus obese children (14, 15). Previous studies have identified pharmacokinetic alterations in obese children including an increase in fat mass compared to lean body mass leading to an altered volume of distribution for certain medications in obese children and additional alterations in hepatic and renal function (19, 20). As a result, obese children may have an increased risk for adverse events if they receive a dose based on their actual body weight versus an adjusted dosing weight like ideal body weight (20). A previous study by Tudela et al. assessed the effect of body mass index on magnesium concentrations in pregnant women with pre-eclampsia (21). They noted that increased body mass index was associated with sub-therapeutic magnesium concentrations, and they hypothesized that it was associated with an increase in volume of distribution in these patients. In aforementioned pharmacokinetic study by Rower et al., they were not able to assess the impact of obesity on magnesium concentrations in children (7).

Five reports including 261 patients (58.4%) evaluated magnesium serum concentrations (13–15, 17, 18). It is important to note that one study by Irazuzta et al. evaluated both ionized and serum magnesium concentrations, while the other reports included assessments of serum magnesium concentrations only (13). Irazuzta and colleagues determined that the positive predictive value of serum and ionized magnesium concentrations was moderate and concluded that serum concentrations did not predict ionized concentrations (13). In most of these reports, the desired serum magnesium concentration was 4–6 mg/dL. Only one study evaluated the magnesium pharmacokinetics and noted that volume of distribution was 0.4 ± 0.13 L/kg with a clearance of 1.58 ± 0.24 mL/kg/min; these data are similar to other studies assessing magnesium pharmacokinetics (7, 14). Out of the 261 patients who had magnesium serum concentrations, only three patients (1.1%) were supratherapeutic with a serum concentration >6 mg/dL, and the authors did not attribute any adverse events to their elevated concentrations (18).

The only studies reporting adverse events attributed to magnesium were noted in those receiving infusions over >24 h (17, 18). Clinically significant adverse events included hypotension (*n* = 74; 16.6%), nausea/vomiting (*n* = 35; 7.8%), mild muscle weakness (*n* = 22; 4.9%), flushing (*n* = 10; 2.2%), and sedation (*n* = 2; 0.4%). For those with hypotension, 170 events occurred in the 74 patients, with most of these episodes associated with diastolic hypotension (*n* = 165; 97.1%) (18). Only five of these patients (6.6%) required an intervention to resolve the hypotension including a fluid bolus (*n* = 2), initiation of intravenous maintenance fluids (*n* = 1), or a decrease in magnesium infusion rate (*n* = 2). The only other adverse event that required intervention was nausea/vomiting; thirty of these patients (*n* = 85.7%) required treatment with ondansetron.

Several practical considerations must be noted when utilizing magnesium sulfate infusions. First, there are limited recommendations for IV concentrations of magnesium sulfate for continuous administration. Magnesium is commercially available as a 50% (500 mg/mL) solution; however, current recommendations are to dilute in dextrose 5 or 0.9% sodium chloride (United States Pharmacopeia) to a usual concentration of 60 mg/mL with a maximum of 200 mg/mL (22). Anecdotally, we currently utilize 40 and 80 mg/mL concentrations; clinicians may need to utilize a more concentrated solution for obese children to minimize volume in patients who may be fluid restricted. Another consideration is related to the drug library for IV smart pump technology for intravenous administration (23). Many institutions may have, in their drug library administration, considerations for intermittent magnesium sulfate boluses for electrolyte replacement. However, their drug library may need to be adjusted to ensure appropriate administration considerations for patients receiving prolonged magnesium sulfate infusions. At this time, due to the limited data pertaining to clinical outcomes, we recommend that the use of prolonged magnesium infusions should be reserved for refractory asthma therapy following other therapies with more robust clinical outcomes, including terbutaline and aminophylline (6). However, if clinicians consider this therapy, given the variability in dosing from these reports, we recommend an initial bolus of magnesium sulfate of 25 mg/kg/dose for those patients with an initial magnesium sulfate concentration <3.5 mg/dL. In addition, we recommend an initial starting dose of 15 mg/kg/h in those <40 kg and 10 mg/kg/h in those >40 kg to achieve a target magnesium sulfate concentration between 4 and 6 mg/dL. Further, we would recommend use of ideal body weight in those ≥2 years of age with a body mass index >95th percentile for weight and sex. We also recommend checking serum magnesium concentrations every 4 h based on the published half-life of approximately 2.5 h and titrate up and down by 5 mg/kg/h to achieve the target magnesium concentrations (7).

In conclusion, there was significant variability in the dosage regimens of those children who received prolonged magnesium sulfate infusions >1 h. Most reports described extended courses of magnesium for ≥4 h for children with refractory asthma treatment. Few reports described the impact of magnesium sulfate on clinical outcomes. Most reports evaluated magnesium serum concentrations and targeted a desired serum concentration between 4 and 6 mg/dL. The only patients who had a documented adverse event were those receiving magnesium >24 h and included hypotension, nausea/vomiting, mild muscle weakness, flushing, and sedation. Based on the limited clinical evidence available, the use of prolonged magnesium sulfate infusions should be reserved as an option for refractory asthma therapy. Future clinical studies should evaluate the difference in clinical outcomes in those who received prolonged magnesium infusions with other therapies (e.g., aminophylline, terbutaline).

## Data Availability Statement

The original contributions presented in the study are included in the article/supplementary material, further inquiries can be directed to the corresponding author.

## Author Contributions

PNJ, ASD, and NG contributed to the conception, writing, and final edits of this manuscript. All authors contributed to the article and approved the submitted version.

## Conflict of Interest

The authors declare that the research was conducted in the absence of any commercial or financial relationships that could be construed as a potential conflict of interest.

## Publisher’s Note

All claims expressed in this article are solely those of the authors and do not necessarily represent those of their affiliated organizations, or those of the publisher, the editors and the reviewers. Any product that may be evaluated in this article, or claim that may be made by its manufacturer, is not guaranteed or endorsed by the publisher.
